# Screening Wild Yeast Isolated from Cocoa Bean Fermentation Using Volatile Compounds Profile

**DOI:** 10.3390/molecules27030902

**Published:** 2022-01-28

**Authors:** Claudia Johanna Sandoval-Lozano, David Caballero-Torres, Luis Javier López-Giraldo

**Affiliations:** Grupo de Investigación en Ciencia y Tecnología de Alimentos (CICTA), Universidad Industrial de Santander (UIS), Bucaramanga 680002, Colombia; davidct.ce@gmail.com

**Keywords:** yeasts, cocoa beans, VCs, GC-FID, PCA, aroma

## Abstract

Yeasts are one of the main ingredients responsible for flavor precursors production associated with sensorial characteristics in chocolate. Using wild yeast isolated from cocoa beans fermentation is emerging as a strategy for developing starter cultures. However, the volatile compounds (VCs) produced by yeasts are not yet known. This study aimed to select wild yeasts with the potential to produce volatile compounds associated with desirable flavor attributes. A total of 150 wild yeasts strains were isolated from the spontaneous cocoa beans fermentation, of which 40 were identified by morphology and physiological features. VCs produced were identified and quantified using SPME-GC-MS and GC-FID and profiles were evaluated statistically by PCA and cluster analysis for the compounds that had a high odor threshold value. Thirty-six VCs produced by these yeasts were identified into six main families, namely esters, alcohols, acids, aldehydes, ketones, and pyrazines. PCA showed the separation of the yeasts into two main clusters. Strains, Y195 and Y246, belong to the first cluster and are the highest producers of alcohols related to floral perceptions. In the second cluster, thirty-three yeasts were grouped by their ability to produce esters. Of all of them, Y110MRS stood out for producing 2-phenyl ethyl acetate and isoamyl acetate associated with fruity perceptions. This screening allowed us to identify yeasts that produced VCs of technological interest and which could be used to develop a starter culture.

## 1. Introduction

Cocoa beans (*Theobroma cacao* L.) are the primary source for the production of chocolate and cocoa products. Cocoa beans’ worldwide production was estimated at 4728 million tons in 2019–2020, of which 18.8% was produced by America [1]. Colombia is considered the 10th producer worldwide, at 63,416 tons of cocoa beans [2]. According to the International Cocoa Organization, there are two main categories for cocoa beans, bulk or ordinary and fine or flavor cocoa [3]. Fine or flavor cocoa is known for its high quality, in particular for its flavor and aroma properties [4]. Between 2018 and 2020 the exporters of cocoa beans increased from 3 to 4 million, approximately [5]. This increased market is due to consumer preferences for healthier chocolate with a single origin and organoleptic properties. As a result, this special cocoa has a differential price compared to the bulk cocoa market; it is estimated that a ton of bulk cocoa has a value between US $2500 and US $2700, while fine or flavor cocoa has values that vary between US $3500 and US $10,000 per ton [6]. Colombia’s cocoa is recognized worldwide for its characteristic taste and aroma [7]; thus, it has the potential to advance the fine and aroma market.

Production of fine or flavor cocoa is influenced by many factors related to the harvest and post-harvest processes, such as genotype, agroclimatic and edaphoclimatic conditions, fermentation, drying, and roasting [8,9,10,11], resulting in a product with variable quality and flavor heterogeneous characteristics. To improve cocoa beans’ sensorial quality from harvest to harvest, one of the main strategies employed is to standardize the cocoa bean fermentation processes using starter cultures. Spontaneous cocoa bean fermentations are mediated by succession with microorganisms that include yeasts, lactic acid, and acetic acid bacterium, filamentous fungi, and spoilage bacteria. Among these microorganisms, yeasts are essentials because they are involved in the conversion of sugars into ethanol as well as aromatic secondary metabolites that may contribute to the flavor of cocoa [8,12].

Therefore, there is a consensus that yeasts are mainly responsible for the production of compounds associated with good sensorial characteristics [13]. Selection of microbial strains, in the cocoa industry, for starter culture is based on the properties thereof, such as tolerance to high ethanol, temperature and acid concentration, resistance to low pH, pectinolytic activity, volatiles compounds production with aroma characteristics, microbial stability, and high fermentation activity [14]. For example, Samagaci et al. [15] isolated 36 yeast pectinolytic strains from spontaneous cocoa beans fermentation that were studied for their response to stress conditions such as high temperature, alcohol, and acid concentration, and low pH. As a result, the authors found that a combination of factors, such as temperature with alcohol or acid, constitute a limiting factor for the growth of pectinolytic yeasts during cocoa bean fermentation. Only strain YS201 was able to develop a response to combined acid and heat shocks. They concluded that the selection of pectinolytic yeasts strains as starter cultures for cocoa fermentation is not an adequate strategy. Similar findings have been reported by Koffi et al. [16], who isolated different strains of high-ethanol producers under physical–chemical stress conditions from spontaneous cocoa fermentation. They found that two strains, *Saccharomyces cerevisiae* YB14, and *Pichia Kudriavzevii* YP13, were able to resist parameters such as temperature, pH, ethanol, and organic acids. Thus, these two strains were best adapted to combined metabolite stress and they may be useable in starter cultures in cocoa fermentation. 

On the other hand, some studies have explored the selection of strains based on volatile compounds production with aroma characteristics. In this context, Koné et al. [13] isolated yeasts associated with spontaneous cocoa fermentation and qualitatively evaluated the aromatic compounds produced by these species. Indeed, the authors identified volatiles compounds produced by *Pichia Kudriavzevii*, *Saccharomyces cerevisiae*, *Galactomyces geotrichum*, and *Wickerhamomyces anomalus*. These yeasts produced a total of 33 aroma compounds grouped into four families such as esters, alcohols, acids, and others, which could produce a great impact on the sensory qualities of cocoa liquor and chocolate. It is important to mention that yeasts produce volatile compounds (VCs) that can enhance sensory characteristics, but they do not prioritize one in particular, which makes it difficult to design culture starters. Meersman et al. [17] selected thermotolerant and high-acetate ester-producing parental *S. cerevisiae* strains for a generation of new hybrids that combine these two properties. The results showed that these hybrids influence the final chocolate flavor, producing aroma-active flavors and limiting the production of undesired off-flavors. Interestingly, their results indicate that the selection or development of high-acetate ester-producing strains can yield fruitier chocolates. Several criteria have been developed to select yeasts for cocoa bean fermentation. However, these strategies are associated with their ability to grow under stress conditions and the production of volatile compounds. Among these strategies, the most representative for the selection of wild yeasts have been the use of volatile compounds, but this approach has been only qualitative, and it is impossible to know if compounds produced by yeast reached the odor-active concentration. In our view, the quantitative assessment of volatile compounds production is needed to identify yeasts with the best potential to produce odor-active compounds. Therefore, we propose the application of PCA to the concentrations of VCs that exceed the detection threshold by each wild yeast, to establish relationships between these variables, which will help choose yeasts that are easy to measure and cost-effective. Based on this strategy, the selected yeasts will employ in future assays to carry out starter cultures that achieve improved fermentation processes. In this context, the present contribution aimed to select wild yeasts with the most potential to produce volatile compounds associated with desirable flavor attributes as well as floral and fruity aromas.

## 2. Results and Discussion

### 2.1. Yeast Isolation

**Figure 1:**
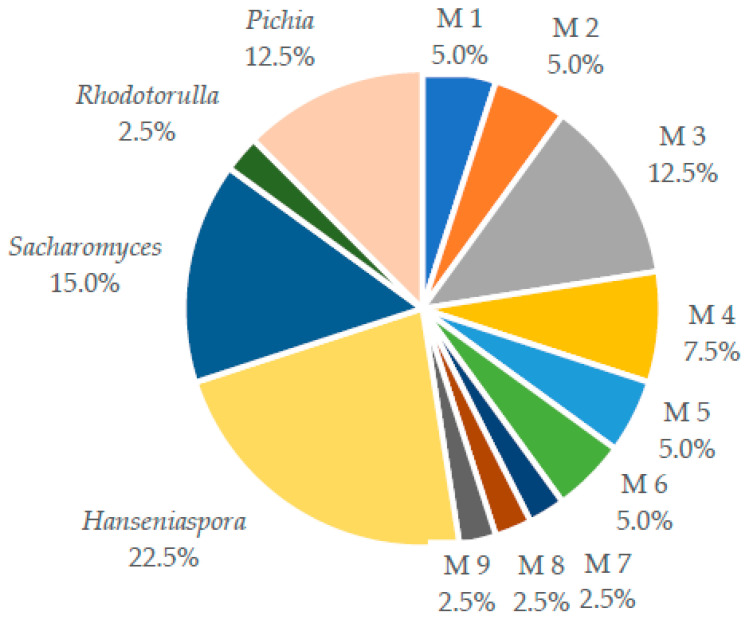
Isolated and identified yeasts from spontaneous cocoa bean fermentations. M1, morphotype 1; M2, morphotype 2; M3, morphotype 3; M4, morphotype 4; M5. morphotype 5; M6, morphotype 6; M7, morphotype 7; M8, morphotype 8; and M9, morphotype 9.

A total of one hundred fifty (150) wild yeast strains were isolated from spontaneous cocoa bean fermentations of three localities in Santander, Colombia. The morphological characterization of yeasts showed thirteen different morphotypes (M) (Figure 1); 52% of the strains (21 isolates) were identified at the genus level, while a total of 48% isolates did not match any of the identities given by RapID Yeast Plus System. Four genera of yeasts were identified: *Hanseniaspora* (*n* = 9), *Saccharomyces* (*n* = 7), *Pichia* (*n* = 5), and *Rhodotorulla* (*n* = 1).

Literature review indicates that yeasts composition during the cocoa fermentation process is directly responsible for developing the flavor and aroma of chocolate. A previous work carried out in the same research group that analyzed the VCs present in different cocoa liquors found 24 compounds produced by yeasts, which suggests that some of the compounds produced during fermentation can be maintained despite the roasting process, for example, 3-methyl butyl acetate and 2-phenylmethyl acetate are also present in cocoa liqueurs. After the roasting process, a greater variety of compounds appear due to the oxidation of alcohols, which decreases their concentration with respect to the fermentation process and give rise to the formation of compounds such as aldehydes and ketones, which they increase their variety and concentration in cocoa liqueurs. Therefore, it is vitally important to know the compounds produced by yeasts that positively impact the quality of the final product in order to better design the starter inoculums [18].

The most frequently reported genera in fermentation cocoa in countries, such as Colombia, Brazil, Cuba, Ghana, Indonesia, Dominican Republic, Malaysia and Mexico are *Saccharomyces*, *Pichia*, *Hanseniaspora*, *Kluyveromyces*, *Hansenula*, *Wickerhamonyces* and, *Candida* [12]. For example, a study conducted by Lozano Tovar et al. [19] identified the species *Wickerhamomyces anomalus*, *Debaryomyces hansenii*, *Meyerozyma guillermondii*, *Pichia guillermondii*, *Pichia Kudriavzevvi*, *Pichia manshurica*, *Trichosporon asahii*, and *Candida parapsilosis* from several cocoa fermentation processes in three regions of Colombia. Their results showed that the strains *W. anomalus*, *D. hansenii*, and *M. guillermondii* were the most promising to be included in a microbial starter culture for cocoa bean fermentation, due to their tolerance of high concentrations of sugars, acidity, and temperature. Experiments lead in Brazil by Magalhaes et al. [20], isolated yeast strains of the genus *Candida*, *Hanseniaspora*, *Rodhotorula*, *Saccharomyces*, *Pichia*, *Saccharomycopsis,* and *Trichosporon* during the fermentation of the three cocoa hybrids (pH 9, pH 15, and pH 16). Their results revealed that *S. cerevisiae* was predominant during fermentation in all experiments, followed by *H. uvarum* and *Pichia*. In addition, Koné et al. [13], isolated *Saccharomyces cerevisiae*, *Candida tropicalis*, *Pichia kudriazevii*, *Pichia galeiforms*, *Galactomyces geotrichum*, and *Wickerhanomyces anomalus*. Amount these yeasts found that *P. kudriazevii*, *S. cerevisiae*, *G. geotrichum*, and *W. anomalus* were the strains that contributed to the formation of cocoa-specific aroma compounds. Therefore, our results are in agreement with previous studies, which confirm that the predominant genera of wild yeast in cocoa fermentation in Colombia are *Saccharomyces*, *Pichia*, *Hanseniaspora*, *Kluyveromyces*, *Hansenula*, *Wickerhamonyces*, and *Torulaspora*. However, to the best of our knowledge, there are no previous reports associated with the concentration of volatile compounds that can be produced by these strains. 

### 2.2. Identification and Quantification for Volatile Compounds (VC’s)

The results related to the identification of VCs for each wild yeast will be exemplified using the yeast Y111. Each compound was tentatively identified using the mass spectra library since standards or chromatographic retention indexes were not available.

**Figure 2:**
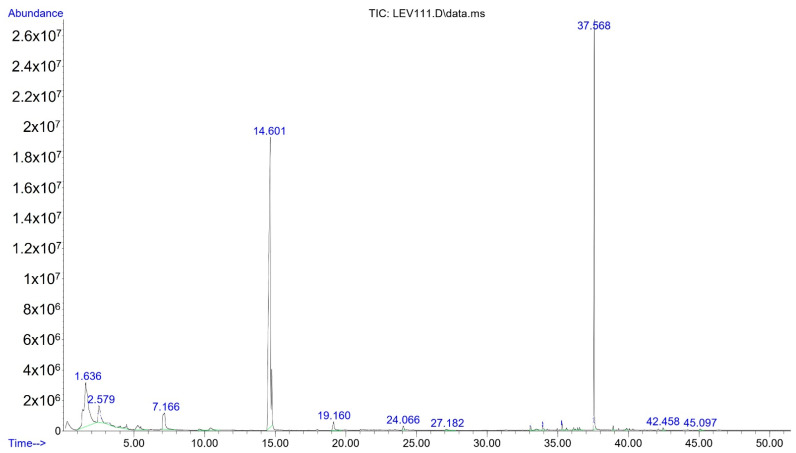
GC-MS chromatogram of the volatile compounds produced by yeast Y111. Peak A is 3-methyl butyl acetate and peak B is 2-phenylmethyl acetate.

**Figure 3:**
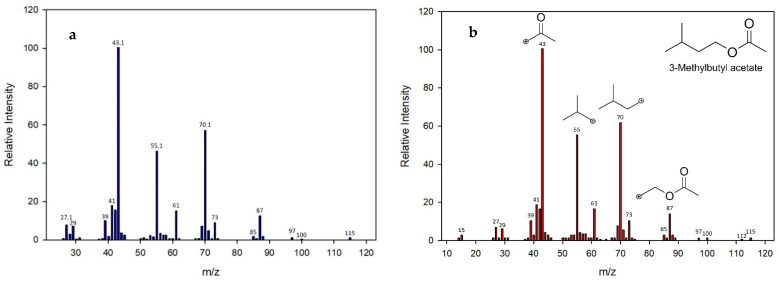
Mass spectra for peak 3-methyl butyl acetate. (**a**) Scan of experimental analysis; (**b**) scan spectrum analysis founded on NIST database and fragmentation pattern.

Figure 2 shows the chromatogram of yeast Y111, where major intense peaks A and B, elute at 14.601 and 37.568 min, respectively. The first peak (A) corresponds tentatively to 3-methyl butyl acetate and the second (B) is tentatively 2-phenylmethyl acetate.; however, Figure 3 shows the mass spectrum for 3-methyl butyl acetate to illustrate the analysis developed. Figure 3a shows the spectrum of the sample while Figure 3b shows the spectrum of NIST SDR 169 libraries from 3-methyl butyl acetate, and a comparison of them figures confirms that both mass spectra coincide with a probability of certainty of 90%. In addition, the fragmentations patterns models of this compound include the following fragments: acetyl at *m*/*z* = 43, isobutyl at *m*/*z* = 55, isoamyl at *m*/*z* = 70 and ethyl acetate at *m*/*z* = 87, which suggests that signal of spectrum probably corresponded to a 3-methyl butyl acetate. Additionally, the identity of the compound was confirmed by comparison of their experimental Kovats Index (KIexp = 859) with reported in the literature for 3-methyl butyl acetate (KI = 867) (Table 1). The absolute relative error between KIexp and KI was 0.9%, therefore, this result confirms the identity of the compound. The results obtained for 3-methyl butyl acetate coincided with those found by Jjunju et al. [21], who confirmed the structure and identity of this compound using the presence of the fragment peak at *m*/*z =* 87.

This procedure was performed for all volatile compounds produced by each wild yeast, obtained a total of thirty-six compounds were tentatively identified and were grouped as follows: 12 esters, 6 alcohols, 6 acids, 5 aldehydes, 5 ketones, and 2 pyrazines (Table 1). For the family of alcohols, the most abundant compounds were ethanol, 3-methyl butanol, and 2-phenyl ethanol (2-PE). Ethanol was the compound that reached the highest concentration of all the volatile compounds evaluated with 974.85 mg/kg, this result is to be expected because ethanol is a primary metabolic product of the fermentation of sugars, which is directly related to the growth of yeasts present in the Sabouraud culture medium. In cocoa fermentation, the ethanol produced by yeasts is used as a carbon source for acetic acid bacteria and triggers biochemical reactions within the cocoa bean that lead to the production of aroma precursors [22]. Results presented in Table 1 show that yeasts were able to produce high concentrations of 2-phenyl ethanol (2-PE), about 51.4509 mg/kg, evidencing the potential of the strains for producing this important aromatic alcohol during the fermentation process. The ability to synthesize this compound naturally has been reported for different strains of yeasts, such as *Saccharomyces cerevisiae*, *Kluyveromyces marxianus*, *Kluyveromyces lactis*, *Pichia fermentans*, *Pichia anomala*, *Schizosaccharomyces pompe,* and *Hansenula anomala*. The compound 2-phenyl ethanol is produced by yeasts using two routes: converting L-phenylalanine (L-Phe) via the Ehrlich pathway or by de novo synthesis from sugars [23,24,25]. According to Fang Y. et al. 2020, alcohols and acids produced by microorganisms during cocoa bean fermentation penetrate the cacao bean and start the chemical reactions that will form the precursor of chocolate’s flavor [11]. Additionally, the alcohols profile is desirable during cocoa fermentation because they are odor-active compounds that denote floral notes. Therefore, the use of wild yeasts that produce higher concentrations of alcohols could have favored the manufacture of flavor cocoa beans with floral notes.

Esters are key compounds in the development of desirable flavors [9,11]. The most abundant compounds found in the esters family were 3-methyl butyl acetate (317.1846 mg/kg), 2-phenyl ethyl acetate (276.7966 mg/kg), and ethyl acetate (102.0740 mg/kg). Between them, the 2-phenyl ethyl acetate (2-PEA) is an interesting phenolic aroma compound in cocoa bean fermentation due to its floral, fruity, and honey-like aroma. The chemical production of 2-PEA is derived from the availability of 2-PE, which is its precursor. This VC is obtained from the esterification of 2-PE with acetic acid and through the transesterification of 2-PE with acetate esters [25]. Amount yeasts, *Hanseniaspora* stands out for being a genus with more efficiency to produce 2-PEA [9,26]. In the case of 3-methyl butyl acetate, its production can be explained by the esterification of 3-methyl butanol by yeasts. On the other than, the synthesis of ethyl- acetate depends on the ethanol concentration. In addition, 3-methyl butyl acetate or isoamyl acetate, 2-phenyl ethyl acetate, and ethyl acetate are considered markers of sensory quality in cocoa fermentation [26] and therefore, the isolated yeasts in this study seem to have esters production potential that improve cocoa beans’ sensorial qualities.

Our results showed the formation of acetic acid, an important contributor to the aroma of unroasted cocoa seeds that were formed during fermentation by action enzymatic degradation of the fruit pulp [27]. Of the pyrazines, two compounds were identified. It is known that pyrazines in cocoa beans are originated from the Strecker degradation in Maillard reactions during the roasting of cocoa beans [22]. However, in this study, two pyrazines were identified in the samples tested. It should be noted that pyrazines are not a family of compounds commonly found during fermentation, however, certain studies have reported that it is possible to find them and they are not exclusively produced during cocoa roasting [18]. 

On the other hand, in the present study three compounds were identified (2-pentanol, α-terpineol, 2,4-di-tert-butylphenol) that had not been reported in similar studies but that correspond to metabolites that can be produced by yeasts. Thus, for example, α-terpineol is a metabolite related to floral aromas associated with lilacs (Table 2). This could be produced thanks to the enzyme monoterpene synthase (MTS) in the early stages of the formation of ergosterol, which is a structural compound of yeast *Saccharomyces cerevisiae* [28]. Indeed a study conducted by King, (2000) revealed the ability of *Saccharomyces cerevisiae, Kluyveromyces lactis* and *Torulaspora delbrueckii* to convert monoterpenoids. These yeasts demonstrated the greatest number of reactions, between them, the conversion of nerol and linalool to α-terpineol [29].

**Table 1:** Volatile compounds concentrations (mg/kg) produced by yeasts quantified by gas chromatography and mass spectrometer (GC–MS) and their odor threshold value.

Families Compounds	Compound	Concentration [mg/kg]		OTV * [mg/kg]
		Min	Max	
alcohols	ethanol	1.9755	974.8454	40
	3-methylbuthanol	0.1687	65.4272	3
	2-pentanol	0.0813	10.2901	4
	2-phenylethanol	0.0759	51.4509	14
	alpha-terpinol	0.0034	2.4455	0.33
	2,4-DI-tert-butylphenol	0.1968	6.3304	-
esters	Ethyl acetate	0.0141	102.0740	5
	2-penthyl acetate	0.2473	14.5378	0.005
	3-methylbuthyl acetate	0.0314	317.1846	0.16
	methylpropil acetate	0.0315	25.1614	1.6
	ethyl benzoate	0.0002	17.3732	0.06
	di-ethyl butanodiate	0.0245	3.8429	200
	ethyl octanoate	0.0094	12.9963	0.58
	2-phenylethyl acetate	0.0081	276.7966	0.65
	ethyl decanoate	0.0104	14.3622	0.2
	ethyl 3-phenylpropanoate	0.0547	12.0306	0.0016
	ethyl 3-phenyl-2-propenoate	0.0355	6.9843	0.0011
	ethyl dodecanoate	0.0150	10.4593	1.5
aldehydes	3-methylbutanal	0.0069	18.6677	0.001
	phenylacetaldehyde	0.0054	54.0838	0.004
	2-phenyl-2-butenal	0.0107	0.1917	1.7
	4-methyl-2-phenyl-2-pentenal	0.0007	2.9953	-
	5-methyl-2-phneyl-2-hexenal	0.0029	3.0785	-
ketones	3-hydroxi-2-butanone	0.0013	0.0603	150
	2-heptanone	0.0125	1.6152	0.14
	2-octanone	0.0077	0.0254	0.05
	2-nonanone	0.0158	0.1291	0.2
	6-methyl-3.5-dihydroxi-2,3-dihydro-4h-pyran-4-one	0.0001	0.3478	0.25
acids	acetic acid	0.0079	76.8848	33
	2 methyl propanoic acid	0.0027	0.0861	2.3
	3-methylbutanoic acid	0.0009	13.0664	0.022
	2-methylbutanoic Acid	0.0125	0.6547	0.05
	decanoic acid	0.4681	3.0440	10
	dodecanoic acid	0.0447	0.3476	-
pyrazines	2-methylpyrazine	6.4451	6.4451	2.5
	2,3,5-trimethylpyrazine	0.0178	0.2596	1.8

* Taken by the authors from: Odor activity values (Aznar et al., 2003 [30]; Cordero et al., 2019 [31]; Cuevas-Glory, Ortiz-Vazquez, Sauri-Duch, and Pino, 2013 [32]; Culleré, Escudero, Cacho, and Ferreira, 2004 [33]; Feng et al., 2018 [34]; Frauendorfer and Schieberle, 2008 [27]; Jetti, Yang, Kurnianta, Finn, and Qian, 2007yang [35]; Liu, Feng, and Chen, 2017 [36]; Ouyang et al., 2017 [37]; Qian and Wang, 2005 [38]).

More of the volatile compounds found in this study were consistent with previous cocoa fermentation studies. In spontaneous cocoa beans fermentation performed in te Ivory Coast were identified yeasts that produced a total of 33 aroma compounds, grouped into esters, alcohols, aldehydes, acids, and ketones. Phenyl ethyl alcohol was the most common VC produced by all yeast [13]. Experiments lead in Brazil by Magalhaes et al. 2017 [20] using different cacao variety clones identified twenty-seven volatile compounds during cocoa fermentation.

Although ethanol’s concentration was the highest while 3-hydroxi-2-butanone concentration was the lowest, these results are insufficient to assure that those compounds’ concentrations reach the levels to be sensed olfactorily; therefore, is very important to analyze this sense. For example, ethanol has an olfactory threshold value (OTV) greater than 3-methyl butyl acetate and 2-phenyl ethyl acetate; which emphasizes that these last compounds are 80 and 17 times easier to perceive than ethanol, respectively. The 3-methyl butanal was the compound identified and quantified with the lowest OTV (0.001 mg/kg), which re der this compound approximately 766 times more perceptible than ethanol. This means that, although the concentration of 3-methy butanal was 52 times lower than of ethanol, in a one-to-one comparison, the characteristic odor of 3-methyl butanal (flowers) will be more perceptible than ethanol. Therefore, the concentrations of compounds are insufficient to perform an equilibrate comparison between different families of compounds responsible for aroma and flavor.

This work stands out for being the first study in which the volatile compounds produced by wild yeasts from Colombian cocoa fermentation boxes are identified and quantified. Additionally, the ability of wild yeasts to produce certain volatile compounds associated with desired aromas in cocoa beans, such as 3-methyl-1-butanol (floral), 3-methyl butyl acetate (banana), acetate of 2-phenyl ethyl (honey or strawberries), phenylacetaldehyde (walnut), among others; compared with other studies such as Meersman’s that used commercial yeasts (Meersman et al., 2016).

In addition, it is observed that the compounds mentioned in the previous paragraph exceed the detection threshold (OTV), therefore, it is very possible that in a directed fermentation, cocoa beans are obtained with the perceptions previously described and complemented in the supplementary material (Table S1).

Although an individual analysis of each yeast and each compound can be done, such an analysis would be extensive due to the large number of analysis possibilities that could be presented. For this reason, in this work, it was decided to propose an approach based on PCA (which includes filters) and cluster analysis to prioritize those yeasts with the greatest potential to produce volatile compounds. 

### 2.3. Principal Component Analysis (PCA)

**Figure 4:**
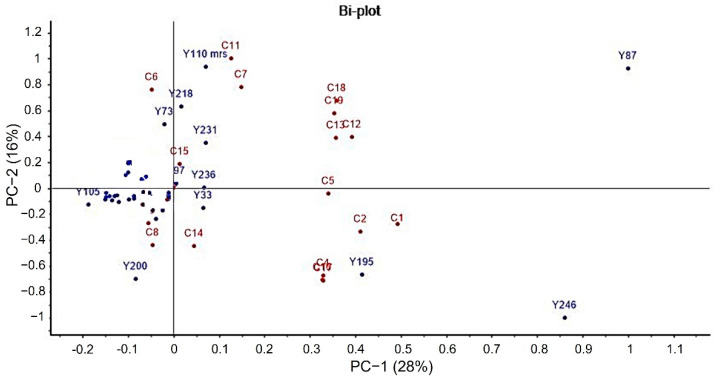
PCA Biplot of volatile compounds produced by yeasts isolated from cocoa pulp-bean mass fermentation of Santander, Colombia. The number of volatile compounds (red) is presented in Table 2. Strains yeast (Blue).

PCA was carried out to visualize the relationship between volatile compounds and yeasts. The biplot shows that the first two principal components explained 44% of the total variance. According to the loading plot (Figure 4), 33 strains of yeasts were characterized by their relationship with fruit perception. The results showed that yeasts, Y218 and Y73, were grouped at the positive PC1 region, and ethyl acetate (C6, pineapple), were associated with this region. Additionally, Y110-mrs yeast was located in positive values of this component, positively correlating with the volatile compounds 2-phenylethyl acetate (C11, fruity, honey) and 3-methylbutyl acetate (isoamyl acetate) (C7, banana). While Y231 was associated with ethyl decanoate (C15, fruity). The presence of these esters has been associated with a fruity perception (Mersman). Ethyl acetate, 2-phenyl ethyl acetate, and 3-methyl butyl acetate (isoamyl acetate) were quantitatively the most prominent acetate esters, and their concentration varied between strains. Of all yeasts, Y110-mrs presented the highest concentrations of 2-phenyl ethyl acetate (317.18 ± 9.69 mg/kg) and 3-methyl butyl acetate (isoamyl acetate (218.03 ± 19.02 mg/kg)).

The strains Y195 and Y246 were placed in the negative PC2 region, and 3-methyl butanol (C2, malty), 2-phenyl ethanol (C4, floral), phenylacetaldehyde (C17, nutty, honey), 3-methyl butanal (C16) were related with floral and sweet perception. Concentrations 51.45 mg/kg of 2-phenyl ethanol (C4, floral), 65.43 mg/kg of 3-methyl butanol (C2, malty), and phenylacetaldehyde (C17, nutty, honey) 12.22 mg/kg were found in Y195. While Y246 reached concentrations of 38.52 mg/kg of 2-phenyl ethanol (C4, floral), 34.92 mg/kg of 3-methyl butanol (C2, malty), and phenylacetaldehyde (C17, nutty, honey) 54.08 mg/kg. Compounds ethyl decanoate (C12, pear, grape, brandy), ethyl 3-phenylpropionate (C13, fruity), and acids were grouped in the positive region of PC1 but they were not related to yeasts.

**Figure 5:**
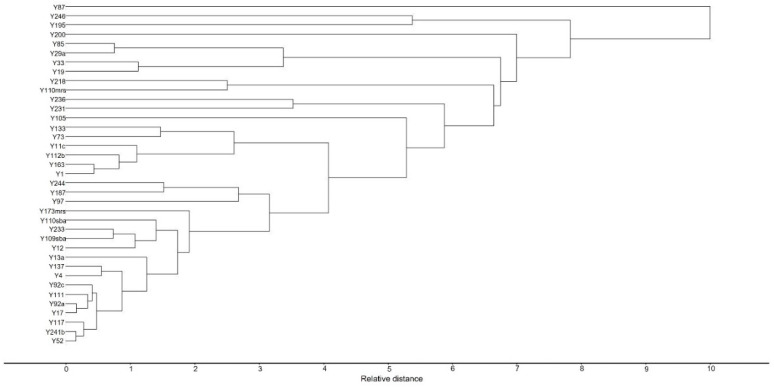
Cluster analysis of cocoa bean yeasts. Strains were grouped based on their VCs production using Ward’s method.

The cluster analysis for yeasts (Figure 5) presented four main groups. Cluster 1 includes strain Y87, which is related to esters, alcohols, and carboxylic acids. Cluster 2 consisted of strains Y195 and Y246, which had the greatest floral contributions. Cluster 3 is constituted by strain Y200, which has the ability to produce 2-phenyl ethanol and methyl propyl acetate, associated with floral and fruity perceptions. The last group (cluster 4) was constituted by strains Y01, Y04, Y11c, Y12, Y13a, Y17, Y19, Y29a, Y33, Y52, Y73, Y85, Y92a, Y92c, Y97, Y105, Y109sba, Y110sba, Y110mrs, Y111, Y112b, Y117, Y133, Y137, Y163, Y173mrs, Y187, Y218, Y231, Y233, Y236, Y214b, and Y244. It should be noted that the majority of yeasts analyzed are found in cluster 4 and these yeasts produced esters frequently associated with fruity perceptions, which agrees with the typical fruity sensorial profile of Colombian cocoa [18].

## 3. Materials and Methods

### 3.1. Spontaneous Cocoa Beans Fermentations

Spontaneous cocoa bean fermentations were carried out in five different farms located in San Vicente de Chucurí (6°55′48.265″ N, 73°24′47.971″79 W), El Carmén de Chucurí (6°50′35.3″ N, 73°27′35.8″ W) and Río Negro (7°20′14.2222″ N, 73°10′41.4552″ W) in the department of Santander, Colombia. Fermentations were done during the harvest season. Cocoa pods of different hybrids were cut from the trees using pruning shears. The pods were opened with a clean machete and the pulp-cocoa beans mass was removed manually. After that, beans were transferred to boxes to ferment, following the conditions shown in Table 3. After fermentation, the cocoa beans were sundried on coverable platforms for a time varying from 4 to 6 days depending on the weather conditions.

### 3.2. Sampling

A typical sample of 100 g was collected, taking 20 g of pulp-cocoa bean mass from five different points inside box fermentation. All samples were taken at 30 cm under the cocoa pulp–bean mass surface. The sampling and measurement of temperatures were done at 0, 6, 12, 18, 24, 36, 48, 72, 96, and 120 h of fermentation. For each time, samples were transferred into sterile plastic polyethylene bags and stored at 4 °C.

### 3.3. Culture-Dependent Microbiological Analysis

A sample of 25 g of pulp-cocoa beans mass from each collected sample was added to 225 mL of sterile saline–peptone water (10 g/L bacteriological peptone (Merck) and 5 g/L NaCl (Merck, Darmstadt, Germany), pH 7.0 ± 0.2). Then, the mixture was manually shaken for 3 min to obtain a cocoa pulp–bean solution. Subsequently, serial dilutions were prepared up to 10^−6^ and aliquots of 100 µL from each dilution were spread by pouring onto inoculated Sabouraud dextrose agar plates (Merck, Darmstadt, Germany) supplemented with 100 mg/L of chloramphenicol (Sigma-Aldrich, Steinheim, Germany). The plates were incubated at 30 °C for 2 days. Isolates were purified through subculturing plating and kept at 4 °C for future identification. 

### 3.4. Yeast Identification

Pure cultures of yeasts were selected and identified visually by their typical morphologies according to established by Kurtzman et al., 2011 [39]. The morphological features observed for each strain were: color, shape, surface, and elevation. Afterward, strains were identified through RapID Yeast Plus System (Remel, Inc., Lexena, KS, USA) and stored in Sabouraud broth with glycerol 20% (*v*/*v*) at −80 °C.

### 3.5. Screening of Yeast Isolates

**Figure 6:**
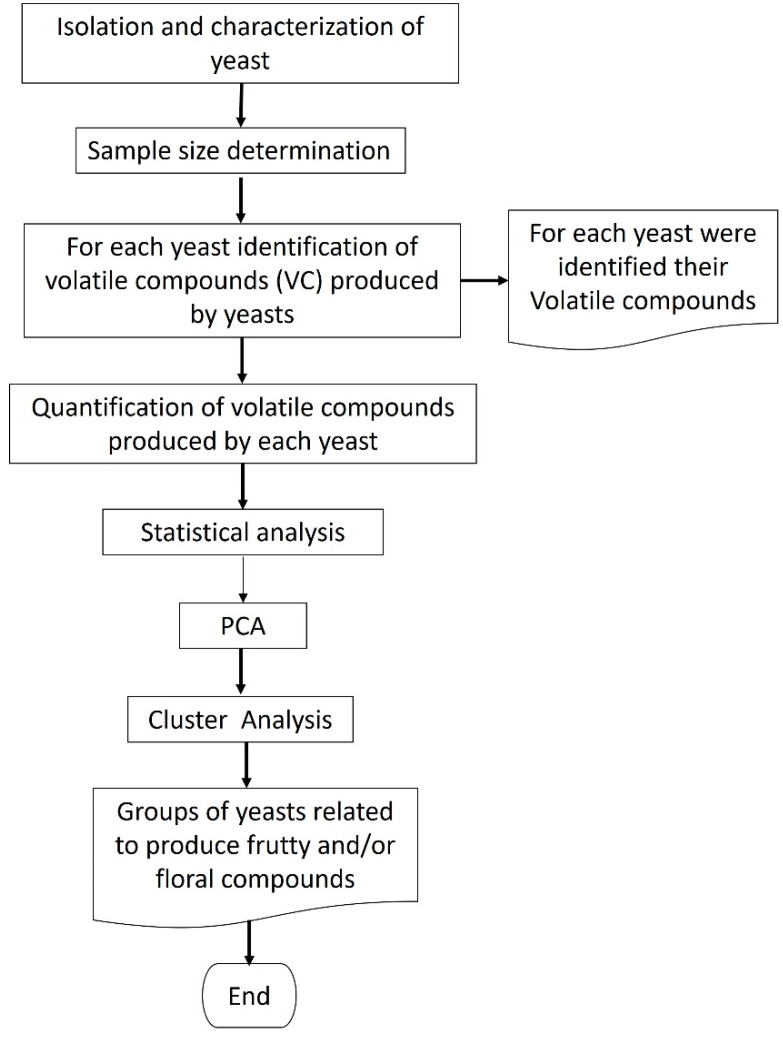
Methodologic flow diagram for yeasts selection according with their volatile compounds fingerprints.

A schematic diagram of the main steps employed for the selection of wild yeasts with the potential to produce VCs is illustrated in Figure 6.

Screening began by fixing the sample size of wild yeast for analysis; for this a population model with a confidence level of 95% and error of 6% [40] was used. Afterward, each wild yeast that conformed to the sample was analyzed for the volatile compounds produced by them, as follows.

#### 3.5.1. Extraction of Volatile Compounds

Volatile compounds (VC) produced by isolated wild yeasts were determined as follows: (i) each isolated wild yeast was incubated at 30 °C, 150 rpm for 12 h using 3 mL Sabouraud broth (Merck); (ii) in the same conditions, vials containing only 3 mL sterile Sabouraud broth were evaluated (control). After that, 2 g of this broth was conserved at 4 °C until volatiles compounds were extracted using the method proposed by Palencia-Blanco et al., 2020 [18]. Briefly, the vial was placed in a water bath at 60 °C for 15 min; then, 50/30-μm fibers of divinylbenzene/carboxene/polydimethylsiloxane (DVB/CAR/PDMS, Supelco, Bellefonte, PA, USA) were exposed into the headspace of the vials for 40 min at 60 °C. Finally, the device that contained the fiber was removed from the vial and the vial was transferred to a GC-injector.

#### 3.5.2. Identification of Volatile Compounds

Volatile compounds adsorbed in microfibers were desorbed using a GC Hewlett-Packard 7890A with an HP 5972 mass selective detector (MSD), Agilent Technologies. Briefly, fibers were exposed for 7 min at 265 °C. Then, the volatile compounds desorbed therefrom were injected into the column using a split mode ratio of 3:1. The temperature gradients used in the oven column were: 30 °C (0–10 min), 3 °C·min^−1^ up to 60 °C, 10 °C·min^−1^ up to 150 °C, 4 °C·min up to 200 °C, 4 °C·min^−1^ up to 250 °C, finally 250 °C (5 min). Separation of compounds was performed in an HP-5 column (30 m × 0.25 mm i.d. × 0.25 μm film thickness). Nitrogen was used as the gas carrier with a constant flow velocity of 1 mL·min^−1^. Later, volatile compounds were analyzed into the mass spectrometer using an electronic impact ionization energy and source temperature of 70 eV and 230 °C, respectively. 

Volatile compounds identification was performed as follows: (i) all compounds with a relative chromatographic area greater than 0.5% were selected for analysis; (ii) mass spectra obtained were compared with the mass spectra of Wiley 275L and NIST SRD69 libraries, and (iii) manual comparisons were performed between the experimental Kovats indexes (KIexp) and the Kovats indexes reported in the literature (KIlit) (Babushok, Linstrom, and Zenkevich, 2011). KI_exp_ for the compounds was calculated using the following equation:

(1) $${\mathrm{KI}}_{\exp}=100n\;+100\left[\frac{t_{\mathrm{Rx}}-t_{\mathrm{Rn}}}{t_{\mathrm{RN}}-t_{\mathrm{Rn}}}\right]$$

where: KI_exp_ = experimental Kovats index of compound x, n = number of carbon atoms of *n*-alkane eluted from compound x, t_Rx_ = retention time of compound x, t_Rn_ y t_RN_ = retention time of *n*-alkanes that eluted before and after compound x, respectively. In this work an *n*-alkanes series varying from Cx to Cy was used.

#### 3.5.3. Quantification of Volatile Compounds Produced by Each Yeast

Quantification of volatile compounds produced by each yeast was performed using the procedure described in Section 3.5.2 with some modifications. A flame ionization detector (FID) was used instead of an MS detector, and hydrogen was used as carrier gas instead of nitrogen. Finally, quantification was performed following the procedure described by Palencia-Blanco et al. [18]. Briefly, 20 µL of toluene at 4200 mg/L was added to 2 mL of methanol as an internal standard (IS). The concentration of volatile compounds was calculated using Equation (2) and response factors (RF) reported by [18] expressed as mg/L. Each sample was evaluated by duplicate.

(2) $$\left[\mathrm{VC}\right]=\frac{\mathrm{RF}\times {\mathrm{Area}}_{\mathrm{VC}}\times \left[\mathrm{IS}\right]}{{\mathrm{Área}}_{\mathrm{SI}}}$$

where, VC: volatile compound concentration; RF: response factor; Area VC: volatile compound area; Area IS: internal standard; and IS: internal standard.

### 3.6. Statistical Data Analysis

For each wild yeast the determination of volatile compound concentration was carried out in duplicate and all results were expressed as an average. Volatile compound concentrations were compared with the odor threshold values (OTVs) from the literature; the main purpose of this comparison was to identify the VCs that had a concentration greater than OTV, and, consequently, could be more important in the potential profile of aromas produced by the wild yeasts.

Two multivariate methods were used to explore the possible contribution that each volatile compound concentration produced by each yeast could have to the aroma. First, principal component analysis was used (PCA) to classify the yeasts according to volatile compounds produced. For this analysis, the data sets were transformed as follows: volatile compounds with an occurrence of less than five times in the entire matrix were eliminated, the same procedure was carried out for yeasts that produced less than five volatile compounds. Then, volatile compound concentrations were converted to Z-scores [17]. The second method performed was a Hierarchical cluster analysis (HCA) using Ward’s minimum variance method and half-squared Euclidean distances. The data were processed with Unscrambler X software (version 10.5.1, CAMO Inc., Oslo, Norway).

## 4. Conclusions

The proposed methodology to select yeasts based on the volatile compounds they produce allowed the concrete prioritization of individuals exhibiting the highest probabilities of producing volatile compounds whose concentrations exceed the odor threshold value (OTV). It also made it possible to classify yeasts according to their tendency to produce floral compounds, such as Y195 and Y246, or yeasts that produce both floral and fruit compounds, such as Y200. This information is key for the development of starter cultures that will improve the sensory characteristics of liqueurs and chocolate products that will be produced using cocoas from targeted fermentation.

## Figures and Tables

**Table 2:** Volatile compounds and their perceptions for the Principal Component Analysis.

Family		Compounds	Attributed Aroma
alcohols	C1	ethanol	alcoholic
	C2	3-methyl butanol Isoamyl alcohol	balsamic fruit
	C3	2-pentanol	green, fruity, sweet, pungent, plastic–floral
	C4	2-phenyl ethanol Phenylethyl alcohol	flowery, rose, spicy, honey-like, caramel
	C5	alpha-terpineol	floral, lilac
esters	C6	ethyl acetate	pineapple
	C7	3-methyl butyl acetate Isoamyl acetate	sweet fruit banana solvent, pear
	C8	methyl propyl acetate	banana, apple
	C9	ethyl Benzoate	fruity, sweet, medicinal, cherry, grape
	C10	ethyl Octanoate	fruity, sweet, musty, floral, pineapple
	C11	2-phenyl ethyl acetate	flowery (rose), honeyfruity, Sweet
	C12	ethyl decanoate	fruity, sweet, pear, grape, brandy
	C13	ethyl 3-phenylpropanoate	fruity
	C14	ethyl 3-phenyl-2-propenoate	fruity, cinnamon
	C15	ethyl dodecanoate	fruity floral, sweet
aldehydes	C16	3-methyl butanal	malty, sweet, chocolate
	C17	phenylacetaldehyde	nutty, honey-like
acids	C18	acetic acid	acid, vinegar
	C19	3-methyl butanoic acid	sweet, rancid

**Table 3:** Spontaneous cocoa beans fermentation conditions on cacao farms of Santander, Colombia.

Farm	Approximate Content *	Opening Pods	Boxes Recubering	Turning	Fermentation Time	Drying Time	Height (msnm)	Relative Humidity (%)	Temperature (°C)
Villa Mónica ^1^	500 kg	10–12 h before start of fermentation	burlap bags	turned daily, starting at 48 h	6 days	6 days	879	77.2	22.4
El Paraiso ^1^	500 kg	10–12 h before start of fermentation	burlap bags	turned daily, starting at 48 h	7 days	6 days	1140	77	23
La Meseta ^1^	400 kg	2 days before start of fermentation	burlap bags	turned daily, starting at 48 h	6 days	6 days	715	77	26
Parcela 7 ^2^	800 kg	2 days before start of fermentation	burlap bags	turned daily, starting at 48 h	7 days	6 days	325	58	30
El Dique ^3^	200 kg	2 days before start of fermentation	burlap bags	turned daily, starting at 48 h	8 days	4 days	1039	80	32

^1^ Farms located in San Vicente de Chucurí, ^2^ El Carmé de Chucurí and ^3^ Río negro. * Aproximate content of pulp-cocoa beans mass in a box fermentation.

## Data Availability

Not applicable.

## Supplementary Materials

The following supporting information can be downloaded online, Table S1: Range of concentrations of volatile compounds produced by yeasts and their corresponding detection limits.

## Sample Availability

Samples of the compounds are not available from the authors.
