# Supplementary material for: Screening Wild Yeast Isolated from Cocoa Bean Fermentation Using Volatile Compounds Profile
Source: Molecules. 2022 Jan 28;27(3):902. doi: 10.3390/molecules27030902 (PMC8838919; doi:10.3390/molecules27030902)
Supplement: Supplementary file 1 [file molecules-27-00902-s001.zip › molecules-1486485-supplementary.pdf]

Table S1. Range of concentrations of volatile compounds produced by yeasts and their corresponding detection limits

Family	Name	Concentration (mg/kg)		OTV [mg/kg]	Yeast 87		Yeast 195		Yeast 200		Yeast 246	
		Min	Max		Con. (mg/kg)	Est. Dev	Con. (mg/kg)	Est. Dev	Con. (mg/kg)	Est. Dev	Con. (mg/kg)	Est. Dev
**Alcohols**	Ethanol	1,9755	974,8454	40	791,736		607,890		164,650		974,845	
	3-methylbuthanol	0,1687	65,4272	3	38,529		65,427				34,919	
	2-pentanol	0,0813	10,2901	4					10,290			
	2-phenylethanol	0,0759	51,4509	14	20,031		51,451		32,603		38,524	
	alpha-terpinol	0,0034	2,4455	0,33	1,559						1,771	
	2,4-DI-Tert-butylphenol	0,1968	6,3304	-	6,330							
**Esters**	Ethyl acetate	0,0141	102,0740	5	-	-	8,466	2,351	-	-	-	-
	2-penthyl acetate	0,2473	14,5378	0,005	13,179	0,487	8,863	0,343	-	-	14,538	1,015
	3-methylbuthyl acetate	0,0314	317,1846	0,16	95,437	15,640	55,069	0,049	-	-	81,755	2,460
	Methylpropil acetate	0,0315	25,1614	1,6	-	-	-	-	25,161	1,317	-	-
	Ethyl Benzoate	0,0002	17,3732	0,06	-	-	0,610	0,383	1,511	0,396	-	-
	Di-ethyl Butanodiate	0,0245	3,8429	200	-	-	-	-	-	-	-	-
	Ethyl Octanoate	0,0094	12,9963	0,58	-	-	-	-	-	-	-	-
	2-phenylethyl acetate	0,0081	276,7966	0,65	130,615	42,178	25,170	9,344	-	-	-	-
	Ethyl Decanoate	0,0104	14,3622	0,2	14,362	8,626	2,783	0,866	-	-	5,548	0,730
	Ethyl 3-Phenylpropanoate	0,0547	12,0306	0,0016	12,031	2,725	-	-	-	-	6,663	0,231
	Ethyl 3-Phenyl-2-propenoate	0,0355	6,9843	0,0011	-	-	3,992	1,469	4,778	3,511	-	-
	Ethyl Dodecanoate	0,0150	10,4593	1,5	-	-	-	-	-	-	-	-
**Aldehydes**	3-methylbutanal	0,0069	18,6677	0,001	-	-	3,947	0,794	-	-	18,668	2,997
	Phenylacetaldehyde	0,0054	54,0838	0,004	-	-	12,219	3,138	-	-	54,084	19,813
	2-phenyl-2-butenal	0,0107	0,1917	1,7	-	-	-	-	-	-	-	-

	4-methyl-2-phenyl-2-pentenal	0,0007	2,9953	-	-	-	-	-	-	-	2,995	0,330
	5-methyl-2-phneyl-2-hexenal	0,0029	3,0785	-	-	-	-	-	-	-	-	-
**Ketones**	3-hydroxi-2-butanone	0,0013	0,0603	150	-	-	-	-	-	-	-	-
	2-heptanone	0,0125	1,6152	0,14	-	-	0,918	0,030	-	-	1,496	0,040
	2-octanone	0,0077	0,0254	0,05	-	-	-	-	-	-	-	-
	2-nonanone	0,0158	0,1291	0,2	-	-	-	-	-	-	-	-
	6-methyl-3,5-dihydroxi-2,3-dihydro-4h-pyran-4-one	0,0001	0,3478	0,25	-	-	0,180	0,041	-	-	-	-
**Acids**	Acetic acid	0,0079	76,8848	33	76,885	3,918	-	-	-	-	1,388	0,120
	2 methyl propanoic acid	0,0027	0,0861	2,3	-	-	-	-	-	-	-	-
	3-methylbutanoic acid	0,0009	13,0664	0,022	13,066	0,347	-	-	-	-	-	-
	2-methylbutanoic Acid	0,0125	0,6547	0,05	-	-	-	-	-	-	-	-
	Decanoic acid	0,4681	3,0440	10	1,054	0,468	3,044	1,032	-	-	-	-
	Dodecanoic acid	0,0447	0,3476	-	-	-	-	-	-	-	-	-
**Pyrazines**	2-methylpyrazine	6,4451	6,4451	2,5	-	-	-	-	6,445	-	-	-
	2,3,5-trimethylpyrazine	0,0178	0,2596	1,8	-	-	-	-	0,023	-	-	-

Concentration matrix of volatile compounds produced by Yeast 01, Yeast 4, Yeast 11c and Yeast 12.

Family	Name	Yeast 01		Yeast 4		Yeast 11c		Yeast 12	
		Con. (mg/kg)	Est. Dev	Con. (mg/kg)	Est. Dev	Con. (mg/kg)	Est. Dev	Con. (mg/kg)	Est. Dev
Alcohols	Ethanol	28,163	-	59,493	-	49,959	-	41,199	-
	3-methylbuthanol	5,456	-	6,651	-	8,664	-	1,381	-
	2-pentanol	-	-	-	-	-	-	-	-
	2-phenylethanol	3,123	-	7,621	-	10,051	-	0,801	-
	alpha-terpinol	-	-	0,252	-	-	-	-	-
	2,4-DI-Tert-butylphenol	0,951	-	-	-	-	-	-	-
Esters	Ethyl acetate	56,392	1,631	18,041	17,018	85,453	7,945	22,093	3,284
	2-penthyl acetate	-	-	-	-	-	-	-	-
	3-methylbuthyl acetate	14,571	1,268	8,905	1,436	9,897	0,600	4,376	0,905
	Methylpropil acetate	-	-	-	-	-	-	-	-
	Ethyl Benzoate	-	-	-	-	0,178	0,000	-	-
	Di-ethyl Butanodiate	-	-	1,312	0,124	2,285	0,025	-	-
	Ethyl Octanoate	-	-	-	-	0,756	0,021	0,698	0,638
	2-phenylethyl acetate	40,116	1,113	2,264	1,418	6,012	0,791	15,387	3,453
	Ethyl Decanoate	-	-	0,322	0,014	1,265	0,265	3,408	2,504
	Ethyl 3-Phenylpropanoate	2,015	0,273	-	-	0,998	0,134	3,238	0,229
	Ethyl 3-Phenyl-2-propenoate	-	-	-	-	0,989	0,106	-	-
	Ethyl Dodecanoate	-	-	-	-	-	-	1,882	0,015
Aldehydes	3-methylbutanal	-	-	-	-	0,287	0,029	-	-
	Phenylacetaldehyde	-	-	0,456	0,219	-	-	-	-
	2-phenyl-2-butenal	-	-	-	-	-	-	-	-
	4-methyl-2-phenyl-2-pentenal	0,895	0,237	-	-	-	-	1,148	0,264
	5-methyl-2-phneyl-2-hexenal	-	-	-	-	-	-	1,038	0,003
Ketones	3-hydroxi-2-butanone	-	-	-	-	0,031	0,001	-	-
	2-heptanone	-	-	-	-	-	-	-	-
	2-octanone	-	-	-	-	-	-	-	-
	2-nonanone	-	-	0,129	0,053	-	-	-	-

	6-methyl-3,5-dihydroxi-2,3-dihydro-4h-pyran-4-one	0,054	0,010	-	-	-	-	-	-
Acids	Acetic acid	-	-	-	-	-	-	-	-
	2 methyl propanoic acid	-	-	-	-	-	-	-	-
	3-methylbutanoic acid	-	-	-	-	0,219	0,006	-	-
	2-methylbutanoic Acid	0,373	0,061	-	-	-	-	-	-
	Decanoic acid	-	-	-	-	-	-	-	-
	Dodecanoic acid	-	-	-	-	-	-	-	-
Pyrazines	2-methylpyrazine	-	-	-	-	-	-	-	-
	2,3,5-trimethylpyrazine	-	-	-	-	-	-	-	-

Matrix of concentrations of volatile compounds produced by Yeast 13a, Yeast 17, Yeast 19 and Yeast 29a.

Family	Name	Yeast 13a		Yeast 17		Yeast 19		Yeast 29a	
		**Con. (mg/kg)**	**Est. Dev**	**Con. (mg/kg)**	**Est. Dev**	**Con. (mg/kg)**	**Est. Dev**	**Con. (mg/kg)**	**Est. Dev**
Alcohols	Ethanol	61,350	-	27,249	-	33,577	-	105,472	-
	3-methylbuthanol	-	-	4,981	-	5,401	-	-	-
	2-pentanol	2,727	-	-	-	-	-	-	-
	2-phenylethanol	1,075	-	1,544	-	2,987	-	1,494	-
	alpha-terpinol	0,084	-	-	-	-	-	-	-
	2,4-DI-Tert-butylphenol	0,656	-	-	-	-	-	-	-
Esters	Ethyl acetate	15,318	1,027	-	-	1,276	0,269	1,457	0,023
	2-penthyl acetate	-	-	-	-	-	-	-	-
	3-methylbuthyl acetate	-	-	-	-	0,766	0,192	1,560	0,137
	Methylpropil acetate	-	-	-	-	-	-	-	-
	Ethyl Benzoate	-	-	-	-	-	-	-	-
	Di-ethyl Butanodiate	-	-	-	-	-	-	-	-
	Ethyl Octanoate	-	-	-	-	12,996	1,360	10,557	0,409
	2-phenylethyl acetate	9,845	0,489	-	-	0,608	0,085	1,820	0,836
	Ethyl Decanoate	0,266	0,106	0,348	0,010	1,952	0,139	-	-
	Ethyl 3-Phenylpropanoate	1,307	0,478	-	-	5,716	0,756	5,344	0,984

	Ethyl 3-Phenyl-2-propenoate	-	-	-	-	6,602	0,693	-	-
	Ethyl Dodecanoate	-	-	-	-	-	-	0,693	0,038
Aldehydes	3-methylbutanal	-	-	-	-	-	-	-	-
	Phenylacetaldehyde	-	-	2,020	0,153	-	-	-	-
	2-phenyl-2-butenal	-	-	-	-	0,192	0,063	-	-
	4-methyl-2-phenyl-2-pentenal	0,444	0,125	-	-	0,820	0,031	-	-
	5-methyl-2-phneyl-2-hexenal	-	-	-	-	-	-	-	-
Ketones	3-hydroxi-2-butanone	-	-	-	-	-	-	-	-
	2-heptanone	-	-	-	-	-	-	-	-
	2-octanone	-	-	-	-	-	-	-	-
	2-nonanone	-	-	-	-	-	-	-	-
	6-methyl-3,5-dihydroxi-2,3-dihydro-4h-pyran-4-one	0,012	0,000	0,060	0,006	-	-	-	-
Acids	Acetic acid	-	-	-	-	-	-	-	-
	2 methyl propanoic acid	-	-	-	-	-	-	-	-
	3-methylbutanoic acid	-	-	-	-	-	-	-	-
	2-methylbutanoic Acid	0,655	0,012	-	-	-	-	-	-
	Decanoic acid	-	-	-	-	-	-	-	-
	Dodecanoic acid	-	-	-	-	-	-	-	-
Pyrazines	2-methylpyrazine	-	-	-	-	-	-	-	-
	2,3,5-trimethylpyrazine	-	-	-	-	0,260	0,050	-	-

Concentration matrix of volatile compounds produced by Yeast 33, Yeast 45, Yeast 47 and Yeast 52.

Family	Name	Yeast 33		Yeast 45		Yeast 47		Yeast 52	
		Con. (mg/kg)	Est. Dev	Con. (mg/kg)	Est. Dev	Con. (mg/kg)	Est. Dev	Con. (mg/kg)	Est. Dev
Alcohols	Ethanol	119,706	-	-	-	-	-	1,975	-
	3-methylbuthanol	8,859	-	-	-	0,532	-	-	-
	2-pentanol	-	-	-	-	-	-	-	-
	2-phenylethanol	4,664	-	0,303	-	0,117	-	-	-
	alpha-terpinol	-	-	-	-	-	-	-	-

	2,4-DI-Tert-butylphenol	-	-	-	-	1,062	-	-	-
Esters	Ethyl acetate	0,681	0,193	-	-	-	-	-	-
	2-penthyl acetate	-	-	-	-	-	-	-	-
	3-methylbuthyl acetate	0,766	0,312	-	-	-	-	-	-
	Methylpropil acetate	0,768	0,232	-	-	-	-	-	-
	Ethyl Benzoate	-	-	-	-	-	-	0,070	0,065
	Di-ethyl Butanodiate	-	-	-	-	-	-	-	-
	Ethyl Octanoate	11,195	2,463	-	-	-	-	-	-
	2-phenylethyl acetate	0,797	0,165	0,360	0,217	12,471	0,315	5,570	0,448
	Ethyl Decanoate	6,924	0,769	-	-	-	-	-	-
	Ethyl 3-Phenylpropanoate	5,488	1,382	-	-	-	-	0,464	0,107
	Ethyl 3-Phenyl-2-propenoate	6,984	0,200	0,660	0,060	-	-	0,292	0,233
	Ethyl Dodecanoate	-	-	-	-	-	-	-	-
Aldehydes	3-methylbutanal	-	-	-	-	-	-	-	-
	Phenylacetaldehyde	-	-	-	-	-	-	-	-
	2-phenyl-2-butenal	-	-	-	-	-	-	-	-
	4-methyl-2-phenyl-2-pentenal	1,092	0,001	-	-	-	-	-	-
	5-methyl-2-phneyl-2-hexenal	-	-	-	-	-	-	-	-
Ketones	3-hydroxi-2-butanone	-	-	-	-	-	-	-	-
	2-heptanone	-	-	-	-	-	-	-	-
	2-octanone	-	-	-	-	-	-	-	-
	2-nonanone	0,080	0,016	-	-	-	-	-	-
	6-methyl-3,5-dihydroxi-2,3-dihydro-4h-pyran-4-one	0,027	0,002	0,029	0,003	0,348	0,073	0,032	0,004
Acids	Acetic acid	-	-	-	-	-	-	-	-
	2 methyl propanoic acid	-	-	-	-	-	-	-	-
	3-methylbutanoic acid	-	-	-	-	-	-	-	-
	2-methylbutanoic Acid	-	-	-	-	-	-	-	-
	Decanoic acid	-	-	-	-	-	-	-	-
	Dodecanoic acid	-	-	-	-	0,348	0,045	-	-
Pyrazines	2-methylpyrazine	-	-	-	-	-	-	-	-
	2,3,5-trimethylpyrazine	0,173	0,018	-	-	-	-	-	-

Concentration matrix of volatile compounds produced by Yeast 73, Yeast 85, Yeast 87 and Yeast 92a.

Family	Name	Yeast 73		Yeast 85		Yeast 87		Yeast 92a	
		**Con. (mg/kg)**	**Est. Dev**	**Con. (mg/kg)**	**Est. Dev**	**Con. (mg/kg)**	**Est. Dev**	**Con. (mg/kg)**	**Est. Dev**
Alcohols	Ethanol	148,228	-	44,079	-	791,736	-	21,912	-
	3-methylbuthanol	2,669	-	11,556	-	38,529	-	3,008	-
	2-pentanol	-	-	-	-	-	-	-	-
	2-phenylethanol	1,028	-	6,879	-	20,031	-	1,952	-
	alpha-terpinol	-	-	-	-	1,559	-	-	-
	2,4-DI-Tert-butylphenol	-	-	-	-	6,330	-	-	-
Esters	Ethyl acetate	67,861	13,035	0,718	0,171	-	-	-	-
	2-penthyl acetate	-	-	-	-	13,179	0,487	-	-
	3-methylbuthyl acetate	210,306	14,868	1,260	0,189	95,437	15,640	-	-
	Methylpropil acetate	-	-	-	-	-	-	-	-
	Ethyl Benzoate	-	-	-	-	-	-	-	-
	Di-ethyl Butanodiate	1,029	0,084	-	-	-	-	-	-
	Ethyl Octanoate	0,251	0,070	11,098	1,553	-	-	-	-
	2-phenylethyl acetate	141,387	8,485	0,988	0,225	130,615	42,178	-	-
	Ethyl Decanoate	2,141	1,105	1,460	1,026	14,362	8,626	0,124	0,069
	Ethyl 3-Phenylpropanoate	1,386	0,303	5,878	1,086	12,031	2,725	-	-
	Ethyl 3-Phenyl-2-propenoate	-	-	-	-	-	-	-	-
	Ethyl Dodecanoate	-	-	-	-	-	-	-	-
Aldehydes	3-methylbutanal	-	-	-	-	-	-	-	-
	Phenylacetaldehyde	-	-	0,268	0,035	-	-	0,170	0,188
	2-phenyl-2-butenal	-	-	-	-	-	-	-	-
	4-methyl-2-phenyl-2-pentenal	0,641	0,135	-	-	-	-	-	-
	5-methyl-2-phneyl-2-hexenal	-	-	-	-	-	-	-	-
Ketones	3-hydroxi-2-butanone	0,060	0,002	-	-	-	-	-	-
	2-heptanone	-	-	-	-	-	-	-	-

	2-octanone	-	-	-	-	-	-	-	-
	2-nonanone	-	-	-	-	-	-	-	-
	6-methyl-3,5-dihydroxi-2,3-dihydro-4h-pyran-4-one	0,033	0,010	-	-	-	-	-	-
Acids	Acetic acid	-	-	-	-	76,885	3,918	-	-
	2 methyl propanoic acid	-	-	-	-	-	-	-	-
	3-methylbutanoic acid	-	-	-	-	13,066	0,347	-	-
	2-methylbutanoic Acid	-	-	-	-	-	-	-	-
	Decanoic acid	-	-	-	-	1,054	0,468	-	-
	Dodecanoic acid	-	-	-	-	-	-	-	-
Pyrazines	2-methylpyrazine	-	-	-	-	-	-	-	-
	2,3,5-trimethylpyrazine	-	-	0,170	0,061	-	-	-	-

Concentration matrix of volatile compounds produced by Yeast 92c, Yeast 97, Yeast 105 and Yeast 109sba.

Family	Name	Yeast 92c		Yeast 97		Yeast 105		Yeast 109 sba	
		Con. (mg/kg)	Est. Dev	Con. (mg/kg)	Est. Dev	Con. (mg/kg)	Est. Dev	Con. (mg/kg)	Est. Dev
Alcohols	Ethanol	25,963	-	131,046	-	42,112	-	70,617	-
	3-methylbuthanol	-	-	4,323	-	0,169	-	5,339	-
	2-pentanol	0,889	-	-	-	2,110	-	-	-
	2-phenylethanol	0,528	-	2,092	-	1,555	-	3,359	-
	alpha-terpinol	-	-	0,286	-	-	-	-	-
	2,4-DI-Tert-butylphenol	0,197	-	0,591	-	0,905	-	-	-
Esters	Ethyl acetate	-	-	31,601	9,218	19,746	0,071	1,847	0,148
	2-penthyl acetate	-	-	-	-	-	-	-	-
	3-methylbuthyl acetate	-	-	5,895	1,076	4,110	0,397	-	-
	Methylpropil acetate	-	-	-	-	-	-	-	-
	Ethyl Benzoate	-	-	0,545	0,016	17,373	3,831	-	-
	Di-ethyl Butanodiate	-	-	-	-	0,252	0,161	-	-
	Ethyl Octanoate	-	-	-	-	1,653	0,365	0,524	0,009
	2-phenylethyl acetate	0,204	0,124	21,213	2,823	-	-	1,842	0,165

	Ethyl Decanoate	0,072	0,039	6,693	2,356	-	-	1,463	0,583
	Ethyl 3-Phenylpropanoate	-	-	1,082	0,750	-	-	3,766	1,041
	Ethyl 3-Phenyl-2-propenoate	-	-	3,519	0,641	0,145	0,049	-	-
	Ethyl Dodecanoate	-	-	2,946	0,059	0,955	0,110	-	-
Aldehydes	3-methylbutanal	-	-	-	-	-	-	-	-
	Phenylacetaldehyde	-	-	-	-	-	-	-	-
	2-phenyl-2-butenal	-	-	-	-	-	-	-	-
	4-methyl-2-phenyl-2-pentenal	-	-	1,612	0,061	0,364	0,053	1,214	0,219
	5-methyl-2-phneyl-2-hexenal	-	-	-	-	-	-	-	-
Ketones	3-hydroxi-2-butanone	-	-	-	-	0,018	0,005	-	-
	2-heptanone	-	-	-	-	-	-	-	-
	2-octanone	-	-	-	-	-	-	-	-
	2-nonanone	-	-	-	-	-	-	-	-
	6-methyl-3,5-dihydroxi-2,3-dihydro-4h-pyran-4-one	-	-	0,196	0,028	-	-	0,083	0,015
Acids	Acetic acid	0,115	0,008	-	-	-	-	-	-
	2 methyl propanoic acid	-	-	-	-	-	-	-	-
	3-methylbutanoic acid	0,038	0,002	-	-	0,048	0,022	-	-
	2-methylbutanoic Acid	-	-	-	-	-	-	-	-
	Decanoic acid	-	-	-	-	-	-	-	-
	Dodecanoic acid	-	-	-	-	-	-	-	-
Pyrazines	2-methylpyrazine	-	-	-	-	-	-	-	-
	2,3,5-trimethylpyrazine	-	-	-	-	-	-	-	-

Concentration matrix of volatile compounds produced by Yeast 110mrs, Yeast 110sba, Yeast 111 and Yeast 112b.

**Family**	**Name**	**Yeast 110 mrs**		**Yeast 110 sba**		**Yeast 111**		**Yeast 112b**	
		**Con. (mg/kg)**	**Est. Dev**	**Con. (mg/kg)**	**Est. Dev**	**Con. (mg/kg)**	**Est. Dev**	**Con. (mg/kg)**	**Est. Dev**
Alcohols	Ethanol	25,315	-	179,763	-	8,359	-	36,057	-
	3-methylbuthanol	0,395	-	3,450	-	0,185	-	3,048	-
	2-pentanol	-	-	-	-	-	-	-	-
	2-phenylethanol	0,220	-	1,748	-	0,076	-	3,147	-

	alpha-terpinol	0,003	-	0,235	-	-	-	-	-
	2,4-DI-Tert-butylphenol	0,531	-	0,376	-	-	-	0,679	-
Esters	Ethyl acetate	102,074	11,216	-	-	4,546	0,782	75,954	10,351
	2-penthyl acetate	-	-	-	-	-	-	-	-
	3-methylbuthyl acetate	317,185	9,691	3,480	0,165	14,806	2,925	11,690	1,802
	Methylpropil acetate	-	-	-	-	-	-	-	-
	Ethyl Benzoate	-	-	0,751	0,043	-	-	0,680	0,119
	Di-ethyl Butanodiate	-	-	-	-	-	-	-	-
	Ethyl Octanoate	-	-	-	-	-	-	0,737	0,024
	2-phenylethyl acetate	218,029	19,022	28,502	0,837	11,358	2,592	45,949	3,989
	Ethyl Decanoate	6,183	1,246	5,223	1,290	0,034	0,013	-	-
	Ethyl 3-Phenylpropanoate	1,960	0,154	-	-	-	-	2,711	0,127
	Ethyl 3-Phenyl-2-propenoate	-	-	-	-	-	-	-	-
	Ethyl Dodecanoate	-	-	-	-	-	-	1,799	0,325
Aldehydes	3-methylbutanal	-	-	1,293	0,007	-	-	-	-
	Phenylacetaldehyde	-	-	-	-	-	-	0,061	0,005
	2-phenyl-2-butenal	-	-	-	-	-	-	-	-
	4-methyl-2-phenyl-2-pentenal	0,836	0,011	1,152	0,254	-	-	1,271	0,056
	5-methyl-2-phneyl-2-hexenal	-	-	-	-	-	-	1,332	0,062
Ketones	3-hydroxi-2-butanone	-	-	-	-	-	-	-	-
	2-heptanone	-	-	-	-	-	-	-	-
	2-octanone	-	-	-	-	-	-	-	-
	2-nonanone	-	-	-	-	-	-	-	-
	6-methyl-3,5-dihydroxi-2,3-dihydro-4h-pyran-4-one	0,196	0,007	0,163	0,033	-	-	-	-
Acids	Acetic acid	16,319	10,988	0,492	2,420	-	-	-	-
	2 methyl propanoic acid	-	-	-	-	0,086	0,003	-	-
	3-methylbutanoic acid	-	-	-	-	-	-	-	-
	2-methylbutanoic Acid	-	-	-	-	-	-	-	-
	Decanoic acid	-	-	-	-	-	-	-	-
	Dodecanoic acid	-	-	-	-	-	-	-	-
Pyrazines	2-methylpyrazine	-	-	-	-	-	-	-	-

	2,3,5-trimethylpyrazine	-	-	-	-	-	-	-	-

Concentration matrix of volatile compounds produced by Yeast 117, Yeast 133, Yeast 137 and Yeast 163.

**Family**	**Name**	**Yeast 117**		**Yeast 133**		**Yeast 137**		**Yeast 163**	
		**Con. (mg/kg)**	**Est. Dev**	**Con. (mg/kg)**	**Est. Dev**	**Con. (mg/kg)**	**Est. Dev**	**Con. (mg/kg)**	**Est. Dev**
Alcohols	Ethanol	-	-	31,688	-	44,244	-	56,947	-
	3-methylbuthanol	0,427	-	-	-	2,462	-	2,090	-
	2-pentanol	0,150	-	1,008	-	-	-	-	-
	2-phenylethanol	2,160	-	0,471	-	1,077	-	0,440	-
	alpha-terpinol	-	-	0,083	-	-	-	0,085	-
	2,4-DI-Tert-butylphenol	-	-	0,257	-	0,892	-	1,114	-
Esters	Ethyl acetate	-	-	28,819	8,222	20,756	1,403	60,334	14,669
	2-penthyl acetate	-	-	-	-	-	-	-	-
	3-methylbuthyl acetate	0,975	0,380	106,224	25,516	3,793	0,031	6,492	0,274
	Methylpropil acetate	-	-	-	-	-	-	-	-
	Ethyl Benzoate	-	-	-	-	-	-	0,567	0,083
	Di-ethyl Butanodiate	-	-	-	-	-	-	-	-
	Ethyl Octanoate	-	-	-	-	-	-	-	-
	2-phenylethyl acetate	-	-	82,067	17,303	10,157	2,161	9,564	0,428
	Ethyl Decanoate	0,113	0,087	0,041	0,030	0,107	0,028	0,151	0,058
	Ethyl 3-Phenylpropanoate	1,507	0,375	1,479	0,055	1,026	0,630	2,573	0,583
	Ethyl 3-Phenyl-2-propenoate	-	-	-	-	-	-	-	-
	Ethyl Dodecanoate	-	-	-	-	-	-	-	-
Aldehydes	3-methylbutanal	-	-	0,253	0,027	-	-	-	-
	Phenylacetaldehyde	-	-	-	-	-	-	-	-
	2-phenyl-2-butenal	-	-	-	-	-	-	-	-
	4-methyl-2-phenyl-2-pentenal	-	-	0,404	0,084	0,326	0,076	0,633	0,030
	5-methyl-2-phneyl-2-hexenal	-	-	-	-	-	-	-	-
Ketones	3-hydroxi-2-butanone	-	-	-	-	-	-	-	-
	2-heptanone	-	-	-	-	-	-	1,615	0,013

	2-octanone	-	-	-	-	-	-	-	-
	2-nonanone	-	-	-	-	-	-	-	-
	6-methyl-3,5-dihydroxi-2,3-dihydro-4h-pyran-4-one	0,018	0,003	0,016	0,003	-	-	-	-
Acids	Acetic acid	-	-	-	-	-	-	-	-
	2 methyl propanoic acid	-	-	-	-	0,043	0,005	-	-
	3-methylbutanoic acid	-	-	-	-	-	-	-	-
	2-methylbutanoic Acid	-	-	-	-	-	-	-	-
	Decanoic acid	-	-	-	-	-	-	-	-
	Dodecanoic acid	-	-	-	-	-	-	-	-
Pyrazines	2-methylpyrazine	-	-	-	-	-	-	-	-
	2,3,5-trimethylpyrazine	-	-	-	-	-	-	-	-

Concentration matrix of volatile compounds produced by Yeast 173mrs, Yeast 187, Yeast 195 and Yeast 200.

**Family**	**Name**	**Yeast 173 mrs**		**Yeast 187**		**Yeast 195**		**Yeast 200**	
		**Con. (mg/kg)**	**Est. Dev**	**Con. (mg/kg)**	**Est. Dev**	**Con. (mg/kg)**	**Est. Dev**	**Con. (mg/kg)**	**Est. Dev**
Alcohols	Ethanol	51,343	-	32,101	-	607,890	-	164,650	-
	3-methylbuthanol	11,508	-	1,773	-	65,427	-	-	-
	2-pentanol	-	-	0,081	-	-	-	10,290	-
	2-phenylethanol	26,641	-	0,582	-	51,451	-	32,603	-
	alpha-terpinol	-	-	0,040	-	-	-	-	-
	2,4-DI-Tert-butylphenol	-	-	1,316	-	-	-	-	-
Esters	Ethyl acetate	2,252	0,344	3,536	0,014	8,466	2,351	-	-
	2-penthyl acetate	-	-	-	-	8,863	0,343	-	-
	3-methylbuthyl acetate	0,890	0,045	48,889	2,018	55,069	0,049	-	-
	Methylpropil acetate	1,038	0,079	14,434	0,235	-	-	25,161	1,317
	Ethyl Benzoate	-	-	1,036	0,276	0,610	0,383	1,511	0,396
	Di-ethyl Butanodiate	-	-	-	-	-	-	-	-
	Ethyl Octanoate	-	-	-	-	-	-	-	-
	2-phenylethyl acetate	1,655	0,782	19,576	1,600	25,170	9,344	-	-
	Ethyl Decanoate	-	-	3,216	0,658	2,783	0,866	-	-

	Ethyl 3-Phenylpropanoate	-	-	-	-	-	-	-	-
	Ethyl 3-Phenyl-2-propenoate	-	-	-	-	3,992	1,469	4,778	3,511
	Ethyl Dodecanoate	-	-	-	-	-	-	-	-
Aldehydes	3-methylbutanal	-	-	-	-	3,947	0,794	-	-
	Phenylacetaldehyde	0,955	0,110	1,225	0,417	12,219	3,138	-	-
	2-phenyl-2-butenal	-	-	-	-	-	-	-	-
	4-methyl-2-phenyl-2-pentenal	2,924	0,438	-	-	-	-	-	-
	5-methyl-2-phneyl-2-hexenal	-	-	-	-	-	-	-	-
Ketones	3-hydroxi-2-butanone	-	-	-	-	-	-	-	-
	2-heptanone	-	-	-	-	0,918	0,030	-	-
	2-octanone	-	-	-	-	-	-	-	-
	2-nonanone	-	-	-	-	-	-	-	-
	6-methyl-3,5-dihydroxi-2,3-dihydro-4h-pyran-4-one	-	-	-	-	0,180	0,041	-	-
Acids	Acetic acid	-	-	1,028	0,092	-	-	-	-
	2 methyl propanoic acid	-	-	-	-	-	-	-	-
	3-methylbutanoic acid	0,345	0,096	-	-	-	-	-	-
	2-methylbutanoic Acid	-	-	-	-	-	-	-	-
	Decanoic acid	-	-	-	-	3,044	1,032	-	-
	Dodecanoic acid	-	-	-	-	-	-	-	-
Pyrazines	2-methylpyrazine	-	-	-	-	-	-	6,445	-
	2,3,5-trimethylpyrazine	-	-	-	-	-	-	0,023	-

Concentration matrix of volatile compounds produced by Yeast 218, Yeast 228, Yeast 231 and Yeast 233.

Family	Name	Yeast 218		Yeast 228		Yeast 231		Yeast 233	
		**Con. (mg/kg)**	**Est. Dev**	**Con. (mg/kg)**	**Est. Dev**	**Con. (mg/kg)**	**Est. Dev**	**Con. (mg/kg)**	**Est. Dev**
Alcohols	Ethanol	197,992	-	27,454	-	77,938	-	54,310	-
	3-methylbuthanol	0,330	-	-	-	14,264	-	1,913	-
	2-pentanol	4,075	-	0,803	-	-	-	-	-
	2-phenylethanol	0,721	-	0,664	-	18,723	-	0,585	-
	alpha-terpinol	-	-	-	-	0,527	-	0,405	-

	2,4-DI-Tert-butylphenol	-	-	-	-	-	-	0,333	-
Esters	Ethyl acetate	49,330	14,654	-	-	78,757	21,847	-	-
	2-penthyl acetate	-	-	-	-	-	-	0,292	0,247
	3-methylbuthyl acetate	248,361	43,971	-	-	27,540	2,732	0,441	0,080
	Methylpropil acetate	-	-	-	-	-	-	0,207	0,032
	Ethyl Benzoate	-	-	-	-	0,609	0,071	-	-
	Di-ethyl Butanodiate	-	-	-	-	3,843	0,353	-	-
	Ethyl Octanoate	2,808	0,086	-	-	-	-	-	-
	2-phenylethyl acetate	276,797	27,968	-	-	140,079	4,353	0,237	0,021
	Ethyl Decanoate	0,618	0,076	-	-	-	-	0,560	0,114
	Ethyl 3-Phenylpropanoate	3,889	0,325	-	-	5,278	0,136	1,723	0,219
	Ethyl 3-Phenyl-2-propenoate	-	-	0,570	0,036	-	-	-	-
	Ethyl Dodecanoate	-	-	-	-	10,459	3,952	-	-
Aldehydes	3-methylbutanal	-	-	-	-	-	-	-	-
	Phenylacetaldehyde	-	-	-	-	-	-	-	-
	2-phenyl-2-butenal	-	-	-	-	-	-	0,076	0,011
	4-methyl-2-phenyl-2-pentenal	0,976	0,015	-	-	1,536	0,051	0,424	0,040
	5-methyl-2-phneyl-2-hexenal	-	-	-	-	3,079	3,062	-	-
Ketones	3-hydroxi-2-butanone	-	-	-	-	-	-	-	-
	2-heptanone	-	-	-	-	-	-	-	-
	2-octanone	-	-	-	-	-	-	-	-
	2-nonanone	-	-	-	-	-	-	-	-
	6-methyl-3,5-dihydroxi-2,3-dihydro-4h-pyran-4-one	0,061	0,007	-	-	-	-	-	-
Acids	Acetic acid	-	-	-	-	-	-	-	-
	2 methyl propanoic acid	-	-	-	-	-	-	-	-
	3-methylbutanoic acid	-	-	-	-	0,455	0,185	-	-
	2-methylbutanoic Acid	-	-	-	-	-	-	-	-
	Decanoic acid	-	-	-	-	-	-	-	-
	Dodecanoic acid	-	-	-	-	-	-	-	-
Pyrazines	2-methylpyrazine	-	-	-	-	-	-	-	-
	2,3,5-trimethylpyrazine	-	-	-	-	-	-	-	-

Concentration matrix of volatile compounds produced by Yeast 236, Yeast 241b, Yeast 224 and Yeast 246.

Family	Name	Yeast 236		Yeast 241b		Yeast 244		Yeast 246	
		Con. (mg/kg)	Est. Dev	Con. (mg/kg)	Est. Dev	Con. (mg/kg)	Est. Dev	Con. (mg/kg)	Est. Dev
Alcohols	Ethanol	45,160	-	-	-	48,805	-	974,845	-
	3-methylbuthanol	2,921	-	0,332	-	5,392	-	34,919	-
	2-pentanol	-	-	-	-	-	-	-	-
	2-phenylethanol	1,146	-	0,759	-	8,438	-	38,524	-
	alpha-terpinol	2,445	-	-	-	0,407	-	1,771	-
	2,4-DI-Tert-butylphenol	0,585	-	-	-	-	-	-	-
Esters	Ethyl acetate	-	-	-	-	1,898	1,603	-	-
	2-penthyl acetate	-	-	-	-	-	-	14,538	1,015
	3-methylbuthyl acetate	1,118	0,324	0,710	0,216	-	-	81,755	2,460
	Methylpropil acetate	-	-	-	-	10,769	3,533	-	-
	Ethyl Benzoate	-	-	0,248	0,169	0,064	0,047	-	-
	Di-ethyl Butanodiate	-	-	-	-	2,732	0,903	-	-
	Ethyl Octanoate	-	-	-	-	-	-	-	-
	2-phenylethyl acetate	0,645	0,159	0,084	0,008	-	-	-	-
	Ethyl Decanoate	0,702	0,609	-	-	0,821	0,562	5,548	0,730
	Ethyl 3-Phenylpropanoate	4,195	0,540	0,848	0,165	1,957	0,806	6,663	0,231
	Ethyl 3-Phenyl-2-propenoate	-	-	-	-	-	-	-	-
	Ethyl Dodecanoate	5,748	1,920	-	-	3,261	1,553	-	-
Aldehydes	3-methylbutanal	-	-	-	-	1,154	0,251	18,668	2,997
	Phenylacetaldehyde	-	-	-	-	-	-	54,084	19,813
	2-phenyl-2-butenal	-	-	-	-	-	-	-	-
	4-methyl-2-phenyl-2-pentenal	1,212	0,642	-	-	0,875	0,247	2,995	0,330
	5-methyl-2-phneyl-2-hexenal	-	-	-	-	-	-	-	-
Ketones	3-hydroxi-2-butanone	-	-	-	-	-	-	-	-
	2-heptanone	-	-	-	-	-	-	1,496	0,040
	2-octanone	0,025	0,008	-	-	-	-	-	-
	2-nonanone	-	-	-	-	-	-	-	-

	6-methyl-3,5-dihydroxi-2,3-dihydro-4h-pyran-4-one	-	-	0,011	0,003	-	-	-	-
Acids	Acetic acid	0,094	0,057	-	-	-	-	1,388	0,120
	2 methyl propanoic acid	-	-	-	-	-	-	-	-
	3-methylbutanoic acid	-	-	0,176	0,079	0,276	0,001	-	-
	2-methylbutanoic Acid	-	-	-	-	-	-	-	-
	Decanoic acid	-	-	-	-	-	-	-	-
	Dodecanoic acid	-	-	-	-	-	-	-	-
Pyrazines	2-methylpyrazine	-	-	-	-	-	-	-	-
	2,3,5-trimethylpyrazine	-	-	-	-	-	-	-	-
